# Impact of the COVID-19 shutdown on orthopedic trauma numbers and patterns in an academic Level I Trauma Center in Berlin, Germany

**DOI:** 10.1371/journal.pone.0246956

**Published:** 2021-02-16

**Authors:** Tazio Maleitzke, Matthias Pumberger, Undine A. Gerlach, Carolin Herrmann, Anna Slagman, Louise S. Henriksen, Frederic von Mauchenheim, Nils Hüttermann, Anabel N. Santos, Florian N. Fleckenstein, Geraldine Rauch, Sven Märdian, Carsten Perka, Ulrich Stöckle, Martin Möckel, Tobias Lindner, Tobias Winkler

**Affiliations:** 1 Center for Musculoskeletal Surgery, Charité –Universitätsmedizin Berlin, Berlin, Germany; 2 Julius Wolff Institute, Charité –Universitätsmedizin Berlin, Berlin, Germany; 3 Berlin Institute of Health (BIH), Berlin, Germany; 4 Division of Emergency and Acute Medicine, Campus Charité Mitte and Virchow-Klinikum, Charité –Universitätsmedizin Berlin, Berlin, Germany; 5 Institute of Biometry and Clinical Epidemiology, Charité –Universitätsmedizin Berlin, Berlin, Germany; 6 Department of Growth and Reproduction, Rigshospitalet, University of Copenhagen, Copenhagen, Denmark; 7 Department of Diagnostic and Interventional Radiology, Charité –Universitätsmedizin Berlin, Berlin, Germany; 8 Berlin Institute of Health Center for Regenerative Therapies, Charité –Universitätsmedizin Berlin, Berlin, Germany; Cedars-Sinai Medical Center, UNITED STATES

## Abstract

**Background:**

The COVID-19 pandemic led to the implementation of drastic shutdown measures worldwide. While quarantine, self-isolation and shutdown laws helped to effectively contain and control the spread of SARS-CoV-2, the impact of COVID-19 shutdowns on trauma care in emergency departments (EDs) remains elusive.

**Methods:**

All ED patient records from the 35-day COVID-19 shutdown (SHUTDOWN) period were retrospectively compared to a calendar-matched control period in 2019 (CTRL) as well as to a pre (PRE)- and post (POST)-shutdown period in an academic Level I Trauma Center in Berlin, Germany. Total patient and orthopedic trauma cases and contacts as well as trauma causes and injury patterns were evaluated during respective periods regarding absolute numbers, incidence rate ratios (IRRs) and risk ratios (RRs).

**Findings:**

Daily total patient cases (SHUTDOWN vs. CTRL, 106.94 vs. 167.54) and orthopedic trauma cases (SHUTDOWN vs. CTRL, 30.91 vs. 52.06) decreased during the SHUTDOWN compared to the CTRL period with IRRs of 0.64 and 0.59. While absolute numbers decreased for most trauma causes during the SHUTDOWN period, we observed increased incidence proportions of household injuries and bicycle accidents with RRs of 1.31 and 1.68 respectively. An RR of 2.41 was observed for injuries due to domestic violence. We further recorded increased incidence proportions of acute and regular substance abuse during the SHUTDOWN period with RRs of 1.63 and 3.22, respectively.

**Conclusions:**

While we observed a relevant decrease in total patient cases, relative proportions of specific trauma causes and injury patterns increased during the COVID-19 shutdown in Berlin, Germany. As government programs offered prompt financial aid during the pandemic to individuals and businesses, additional social support may be considered for vulnerable domestic environments.

## Introduction

Since first reports of respiratory tract infections due to a novel coronavirus in late 2019 the rapid spread of SARS-CoV-2 has led to a global health pandemic with vast impacts on society, economy and healthcare systems [1, 2]. As a result, governments implemented drastic shutdown measures to slow-down infection rates and contain the disease outbreak. While some communities are slowly returning to normality, increasing infection numbers in the United States of America and India during the summer and fall of 2020 are proof that the fight against COVID-19 has not yet been won [3, 4].

On March 16^th^ 2020, the German government and the federal states announced a temporary shutdown of major social institutions, including schools, universities, restaurants, bars, theaters, non-essential businesses and nightclubs. Social contacts were restricted, following a one-household-plus-one rule, and the shutdown was maintained for a little over a month. On April 20^th^ 2020, Berlin officials started gradually re-opening small businesses, restaurants, schools and other institutions with new regulations including the use of face masks in most indoor public spaces.

In combination with nationwide shutdowns, quarantine and isolation led to fundamental shifts in daily routines of individuals and businesses by an unforeseen magnitude. Comparable deprivations of social and material resources have previously only been observed after natural disasters. After Hurricane Katrina in 2005 [5] and the tsunami disaster in Sri Lanka in 2004 [6], intimate partner violence increased significantly and was reported to prevail for up to a year after the events [7]. Reasons for the rise in violence included increased emotional stress, unemployment, reduced income and increased substance abuse [8, 9].

Scientific evaluations of trauma and injury patterns in emergency departments (EDs) during the COVID-19 pandemic are still scarce and only slowly emerging [10–13], yet credible news outlets and United Nations reports have pointed out a rise in domestic violence [14, 15]. While potential psychological trauma due to the COVID-19 pandemic has recently been summarized [16], data on physical trauma is urgently needed. Testing facilities at hospitals proved to be highly frequented by patients and healthcare staff during the SARS-CoV-2 pandemic [17]. Yet, due to fear of uncontrolled in-hospital exposure to the virus, subsequent self-treatment of minor injuries and limited practice of harmful behavior (e.g. sports, driving), overall ED admissions and trauma patterns may have been altered during the COVID-19 pandemic.

In this study we evaluated total and daily numbers of patient cases and contacts treated in an academic Level I Trauma Center ED in Berlin, Germany, during the 35-day COVID-19 shutdown (SHUTDOWN) and a calendar-matched control period in 2019 (CTRL). Trauma causes and injury patterns of orthopedic trauma patients during the SHUTDOWN were compared to the CTRL period. Additionally, the number of daily cases and contacts were compared to a shutdown-preceding 35-day transition (PRE) and shutdown-succeeding transition period (POST) in 2020.

## Methods

### Study design and setting

**Fig 1:**
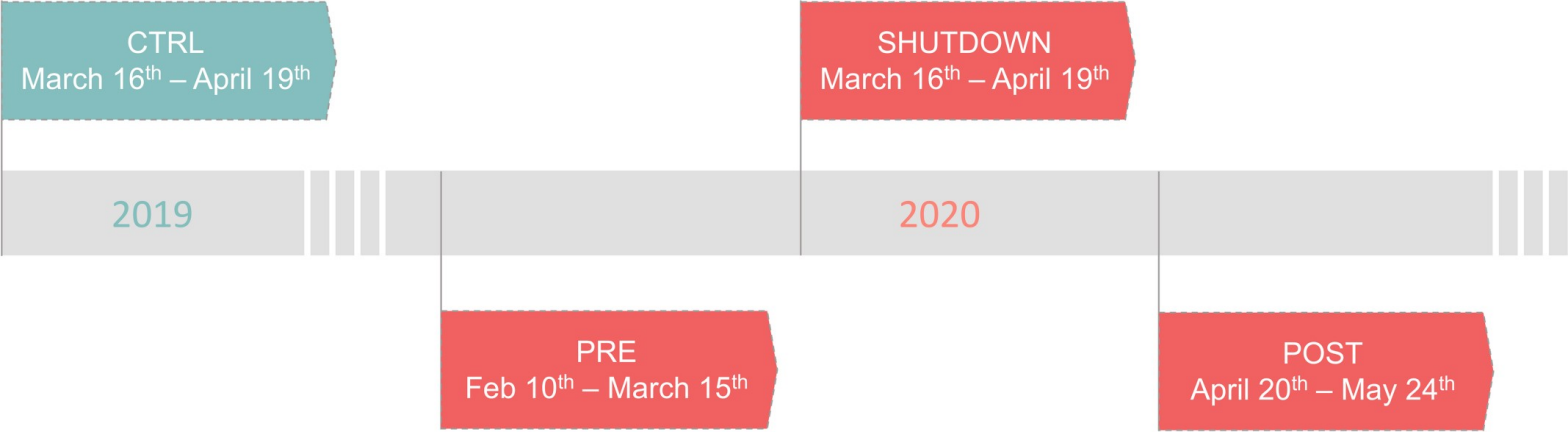
Longitudinal time chart of analyzed time frames before, during and after the COVID-19 shutdown in Berlin, Germany. CTRL = Control; PRE = pre shutdown transition; POST = post shutdown transition; SHUTDOWN = shutdown.

All medical records of patients admitted to our central ED during the following periods were retrospectively evaluated: March 16^th^ until April 19^th^ 2019 and February 10^th^ until May 24^th^ 2020. We included all patients who sought medical care at the ED regardless of whether they were admitted, transferred or directly discharged following acute treatment. To compare periods with different social distancing restrictions, according to the German Infection Protection Act, we defined the following groups:

- CTRL period: March 16^th^–April 19^th^ 2019 (35 days);
- PRE period: February 10^th^–March 15^th^ 2020 (35 days);
- SHUTDOWN period: March 16^th^–April 19^th^ 2020 (35 days);
- POST period: April 20^th^–May 24^th^ 2020 (35 days) (Fig 1).

### General patient volumes

Our Level I Trauma Center is one of five in Berlin, Germany, serving a community of approximately 3.7 million people. 63,394 patients were treated in our ED in 2019, and 18,038 of these were categorized as orthopedic trauma cases.

### Measurements and specifics

Absolute and daily average values were calculated for ED patient cases and contacts. Patient cases were defined as the number of patients who presented to the ED during aforementioned time periods. Patient contacts were defined as the number of medical specialties that were consulted per case. Example: A patient presenting with a distal radius fracture and shortness of breath was seen by both, an orthopedic trauma surgeon and an internal medicine physician, resulting in two patient contacts for this case. Ethical approval was obtained from the local hospital ethics committee (Ethikkommission der Charité –Universitätsmedizin Berlin: EA1/082/20). Trauma calls included trauma team activation for suspected multiple trauma, coma and aortic dissection. All orthopedic trauma patients self-identified as female or male, therefore no additional genders were reported.

### Statistical analyses

**Fig 2:**
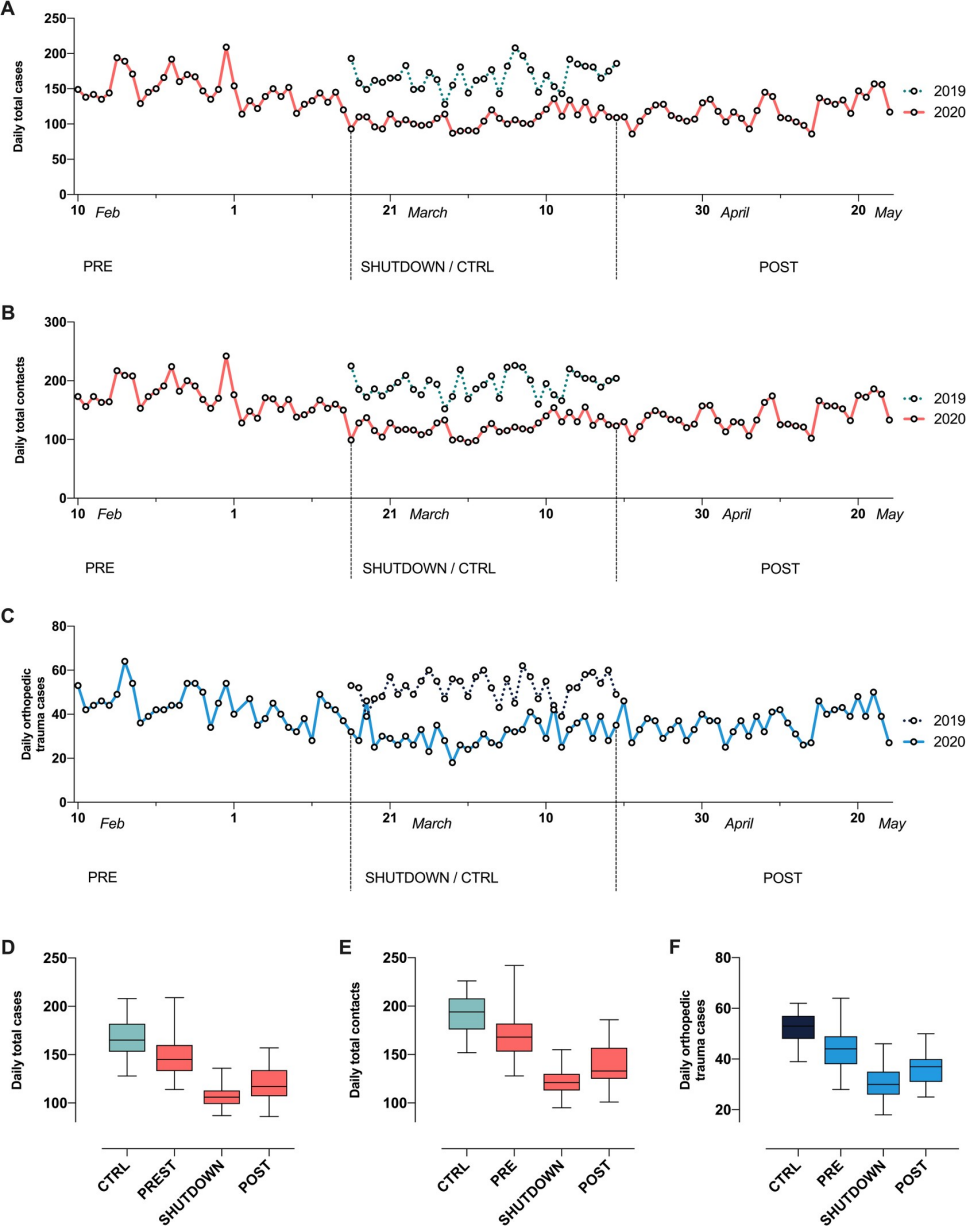
Daily total cases, contacts and orthopedic trauma cases before, during and after the COVID-19 shutdown. A–C: Number of daily (A) total cases, (B) total contacts and (C) orthopedic trauma cases day by day during the four defined periods. Dotted lines indicate the CTRL period in 2019, solid lines indicate PRE, SHUTDOWN and POST periods in 2020. D–F: Box-whisker plots show the number of daily (D) total cases, (E) total contacts and (F) orthopedic trauma cases during the four different periods. CTRL = control; PRE = pre shutdown transition; POST = post shutdown transition; SHUTDOWN = shutdown.

Daily total cases, daily total contacts and daily orthopedic trauma cases and their standard deviations (SDs) were calculated. Daily numbers were plotted as a calendar time function for the CTRL (2019) as well as for the PRE, SHUTDOWN and POST (2020) periods (Fig 2A–2C). Boxplots were created for the same periods (Fig 2D–2F). Upper and lower whiskers represent the respective minimum and maximal values.

Average daily numbers of ED patient cases and contacts were compared between different time periods by means of incidence rate ratios (IRRs). Incidence rates were determined as the cumulative case numbers per period divided by the number of days in the corresponding time periods. IRRs and corresponding 95% confidence intervals (CIs) for comparison of different periods were determined by negative binomial regression analysis. For the explorative design of the study, the 95% CIs were not adjusted for multiple testing and p-values were not reported for that reason.

Furthermore, for orthopedic trauma cases we reviewed different pre-selected variables (S1 File) from ED charts and analyzed changes between the SHUTDOWN and CTRL period. We focused on changes of incidence proportions of the pre-selected variables. Incidence proportions were defined as cumulative numbers of cases of specific pre-selected variables including trauma causes and injury patterns divided by the cumulative number of orthopedic trauma cases within respective periods. To compare incidence proportions, we calculated risk ratios (RR) and related 95% CIs. Again, confidence levels were not adjusted for multiple testing due to the explorative design of the study. All statistical analyses were conducted in R version 4.0.0 and R Studio version 1.2.5001 [18]. RRs and 95% CIs were calculated using the “*riskratio”* function in the R package “epitools” [19].

## Results

### Overall patient cases, contacts and orthopedic trauma cases

The total number of ED patient cases during the 35-day SHUTDOWN period was 3743 and 5864 during the calendar-matched CTRL period. The total number of patient contacts decreased from 6762 during the CTRL to 4255 during the SHUTDOWN period and a similar pattern was seen for daily orthopedic trauma cases during the CTRL (n = 1822) and SHUTDOWN period (n = 1082). Daily numbers decreased for all three parameters during the PRE compared to the CTRL period and during the SHUTDOWN compared to the PRE period. Finally, an increase in daily numbers was observed during the POST compared to the SHUTDOWN period, again for all three parameters. Total and daily numbers as well as IRRs are summarized in Fig 2 and Table 1.

**Table 1:** Numbers (total and daily) of cases, contacts and orthopedic trauma cases as well as IRRs for the four defined periods (CTRL, PRE, SHUTDOWN, POST) before, during and after the COVID-19 shutdown in Berlin, Germany.

	CTRL (35 d)	PRE (35 d)	SHUTDOWN (35 d)	POST (35 d)
Total cases (n)	5864	5200	3743	4176
Daily total cases (mean (SD))	167.5 (18.1)	148.6 (22.5)	106.9 (12.2)	119.3 (18.3)
IRR [95% CI]	SHUTDOWN vs. CTRL	PRE vs. CTRL	SHUTDOWN vs. PRE	POST vs. SHUTDOWN
	**0.64** [0.61; 0.67]	**0.89** [0.84; 0.94]	**0.72** [0.68; 0.77]	**1.12** [1.05; 1.19]
Total contacts (n)	6762	5998	4255	4898
Daily total contacts (mean (SD))	193.2 (19.8)	171.4 (26.0)	121.6 (15.1)	139.9 (22.4)
IRR [95% CI]	SHUTDOWN vs. CTRL	PRE vs. CTRL	SHUTDOWN vs. PRE	POST vs. SHUTDOWN
	**0.63** [0.60; 0.66]	**0.89** [0.84; 0.94]	**0.71** [0.67; 0.76]	**1.15** [1.08; 1.23]
Orthopedic trauma cases (n)	1822	1573	1082	1266
Daily orthopedic trauma cases (mean (SD))	52.1 (6.0)	44.9 (10.9)	30.9 (6.1)	36.2 (6.6)
IRR [95% CI]	SHUTDOWN vs. CTRL	PRE vs. CTRL	SHUTDOWN vs. PRE	POST vs. SHUTDOWN
	**0.59** [0.55; 0.64]	**0.86** [0.80; 0.94]	**0.69** [0.62; 0.76]	**1.17** [1.07; 1.28]

All RRs with a CI not including 1 are highlighted in bold. 95% CI = 95% confidence interval; CTRL = control; ED = emergency department; IRR = incidence rate ratio; PRE = pre shutdown transition; POST = post shutdown transition, SD = standard deviation; SHUTDOWN = shutdown.

To assess potential changes in demographics, trauma causes and injury patterns of orthopedic trauma patients during the COVID-19 shutdown, the development of absolute numbers and incidence proportions during the 35-day SHUTDOWN were evaluated and compared to a calendar-matched 35-day CTRL period. For further relative assessments of data, RRs were calculated with respect to overall orthopedic trauma patient cases during respective periods. Absolute numbers, incidence proportions and RRs are displayed in detail in Tables 2 and 3.

**Table 2:** Summarized demographics, trauma environments, trauma causes, non-traumatic orthopedic presentations, treatment and discharge metrics before and during the COVID-19 shutdown in Berlin, Germany.

	CTRL (35 d) with total n (incidence proportion: n/1822)	SHUTDOWN (35 d) with total n (incidence proportion: n/1082)	RR SHUTDOWN vs. CTRL [95% CI]
All	1822 (1)	1082 (1)	-
**Gender**			
Female	805 (0.44)	451 (0.42)	0.94 [0.86; 1.03]
Male	1017 (0.56)	631 (0.58)	1.04 [0.98; 1.11]
**Trauma calls, substance abuse and homelessness**			
Trauma calls	88 (0.05)	71 (0.07)	1.36 [1.00; 1.84]
Deaths <24 hours	6 (0.003)	4 (0.004)	0.32 [0.32; 3.97]
Deaths >24 hours	5 (0.003)	1 (0.001)	0.34 [0.04; 2.88]
Acute intoxications*	87 (0.05)	79 (0.07)	**1.53 [1.14; 2.05]**
Alcohol intoxications	68 (0.04)	66 (0.06)	**1.63 [1.17; 2.27]**
Other intoxications	14 (0.01)	9 (0.01)	1.08 [0.47; 2.49]
Regular substance abuse	35 (0.02)	67 (0.06)	**3.22 [2.16; 4.82]**
Homeless	19 (0.01)	23 (0.02)	**2.04 [1.12; 3.73]**
**Accidents/injuries in private environments**			
Household accidents	340 (0.19)	264 (0.24)	**1.31 [1.13; 1.51]**
Sport accidents	94 (0.05)	25 (0.02)	**0.45 [0.29; 0.69]**
Nightlife-related accidents	15 (0.01)	6 (0.01)	0.67 [0.26; 1.73]
Self-harm	20 (0.01)	17 (0.02)	1.43 [0.75; 2.72]
Suicide attempts	2 (0.001)	3 (0.003)	2.53 [0.42; 15.09]
Assault-related injuries	83 (0.05)	59 (0.05)	1.20 [0.86; 1.66]
Robbery-related injuries	6 (0.003)	3 (0.003)	0.84 [0.21; 3.36]
Domestic violence-related injuries	14 (0.01)	20 (0.02)	**2.41 [1.22; 4.47]**
**Traffic and workplace accidents/injuries**			
Overall traffic accidents	169 (0.09)	110 (0.10)	1.10 [0.87; 1.38]
Pedestrian accidents	65 (0.04)	27 (0.02)	0.70 [0.45; 1.09]
Bicycle accidents	47 (0.03)	47 (0.04)	**1.68 [1.13; 2.51]**
Motor vehicle accidents	40 (0.02)	32 (0.03)	1.35 [0.85; 2.13]
Public transport accidents	17 (0.01)	4 (0.004)	0.40 [0.13; 1.17]
Workplace accidents	163 (0.09)	120 (0.11)	1.24 [0.99; 1.55]
Way to/from workplace accidents	58 (0.03)	16 (0.01)	**0.46 [0.27; 0.80]**
Workplace violence-related injuries	8 (0.004)	12 (0.01)	**2.53 [1.04; 6.16]**
**Non-traumatic orthopedic presentations**			
Overall non-traumatic orthopedic cases**	678 (0.37)	356 (0.33)	**0.88 [0.80; 0.98]**
Unspecific pain	255 (0.14)	104 (0.10)	**0.69 [0.55; 0.85]**
Low back pain	91 (0.05)	42 (0.04)	0.78 [0.54; 1.11]
Local infections	72 (0.04)	44 (0.04)	1.03 [0.71; 1.49]
Check-up visits	55 (0.03)	29 (0.03)	0.89 [0.57; 1.38]
Internal medicine referrals	146 (0.08)	91 (0.08)	1.05 [0.82; 1.35]
**Treatment**			
Conservative treatment	1412 (0.77)	757 (0.70)	**0.90 [0.86; 0.95]**
Minor ED surgery	281 (0.15)	250 (0.23)	**1.50 [1.29; 1.75]**
(Semi-)elective surgery	103 (0.06)	55 (0.05)	0.90 [0.65; 1.24]
Emergency surgery	26 (0.01)	20 (0.02)	1.30 [0.73; 2.31]
**Admissions and discharges**			
Discharged from ED/transferred	1344 (0.74)	835 (0.77)	1.05 [1.00; 1.09]
Admitted	478 (0.26)	247 (0.23)	**0.87 [0.76; 0.99]**
Discharged <24 hours	212 (0.12)	93 (0.09)	**0.74 [0.59; 0.93]**
Discharged <7 days	179 (0.10)	115 (0.10)	1.08 [0.87; 1.35]
Discharged <1 month	75 (0.04)	36 (0.03)	0.81 [0.55; 1.19]
Discharged >1 month	12 (0.07)	3 (0.003)	0.42 [0.12; 1.49]

Total numbers and incidence proportions are shown in the first two columns for CTRL and SHUTDOWN periods and RRs for SHUTDOWN vs. CTRL in the third. 1822 and 1082 are the total numbers of orthopedic trauma cases in CTRL and SHUTDOWN periods, respectively. All RRs with a CI not including 1 are highlighted in bold. 95% CI = 95% confidence interval; CTRL = control; RR = risk ratio; SHUTDOWN = shutdown.

* The value for acute intoxications is a result of the sum of alcohol intoxications, other intoxications (incl. narcotics, amphetamines, cannabis, etc.) and unknown intoxications (not shown).

** The number of overall non-traumatic orthopedic cases results from the sum of unspecific pain, low back pain, local infections, check-up visits, internal medicine referrals and dermatology referrals (not shown); gynecological referrals (not shown), etc.

**Table 3:** Total numbers, incidence proportions and RRs of different fractures and intracranial hemorrhages before (CTRL) and during the COVID-19 shutdown (SHUTDOWN) in Berlin, Germany.

	CTRL (35 d) with total n (incidence proportion: n/1822)	SHUTDOWN (35 d) with total n (incidence proportion: n/1082)	SHUTDOWN vs. CTRL RR [95% CI]
All fractures	339 (0.19)	241 (0.22)	1.20 [1.03; 1.39]
Facial fractures	68 (0.04)	35 (0.03)	0.87 [0.58; 1.29]
Radius/ulna fractures	38 (0.02)	29 (0.03)	1.29 [0.80; 2.07]
Hand fractures	34 (0.02)	20 (0.02)	0.99 [0.57; 1.71]
Femoral fractures	31 (0.02)	17 (0.02)	0.92 [0.51; 1.66]
Skull fractures	26 (0.01)	12 (0.01)	0.78 [0.39; 1.53]
Foot fractures	20 (0.01)	9 (0.01)	0.76 [0.35; 1.66]
Rib fractures	15 (0.01)	15 (0.01)	1.68 [0.83; 3.43]
Tibia/fibula fractures	15 (0.01)	12 (0.01)	1.35 [0.63; 2.87]
Thoracic spine fractures	13 (0.01)	9 (0.01)	1.17 [0.50; 2.72]
Humerus fractures	12 (0.01)	22 (0.02)	**3.09 [1.53; 6.21]**
Pelvic/sacral fractures	12 (0.01)	5 (0.01)	0.70 [0.25; 1.99]
Lumbar spine fractures	10 (0.01)	11 (0.10)	1.85 [0.79; 4.35]
Clavicle fractures	7 (0.004)	9 (0.01)	2.17 [0.81; 5.80]
Cervical spine fractures	6 (0.003)	5 (0.003)	1.40 [0.43; 4.59]
Patella fractures	1 (0.001)	2 (0.002)	3.37 [0.31; 37.10]
Open fractures	11 (0.01)	4 (0.004)	0.61 [0.20; 1.92]
Patients with intracranial hemorrhages	18 (0.01)	19 (0.02)	1.78 [0.94; 3.37]

1822 and 1082 are the total numbers of orthopedic trauma cases in CTRL and SHUTDOWN periods, respectively. All RRs where the 95% CI does not include 1 are highlighted in bold. 95% CI = 95% confidence interval; CTRL = control; RR = risk ratio; SHUTDOWN = shutdown.

### Gender, trauma calls, substance abuse and homelessness

Absolute numbers of female and male patients declined during the SHUTDOWN compared to the CTRL period without any difference in gender distribution on a relative scale. Trauma calls and trauma related deaths in the ED <24 hours and >24 hours showed no relative differences during the SHUTDOWN compared to the CTRL period.

**Fig 3:**
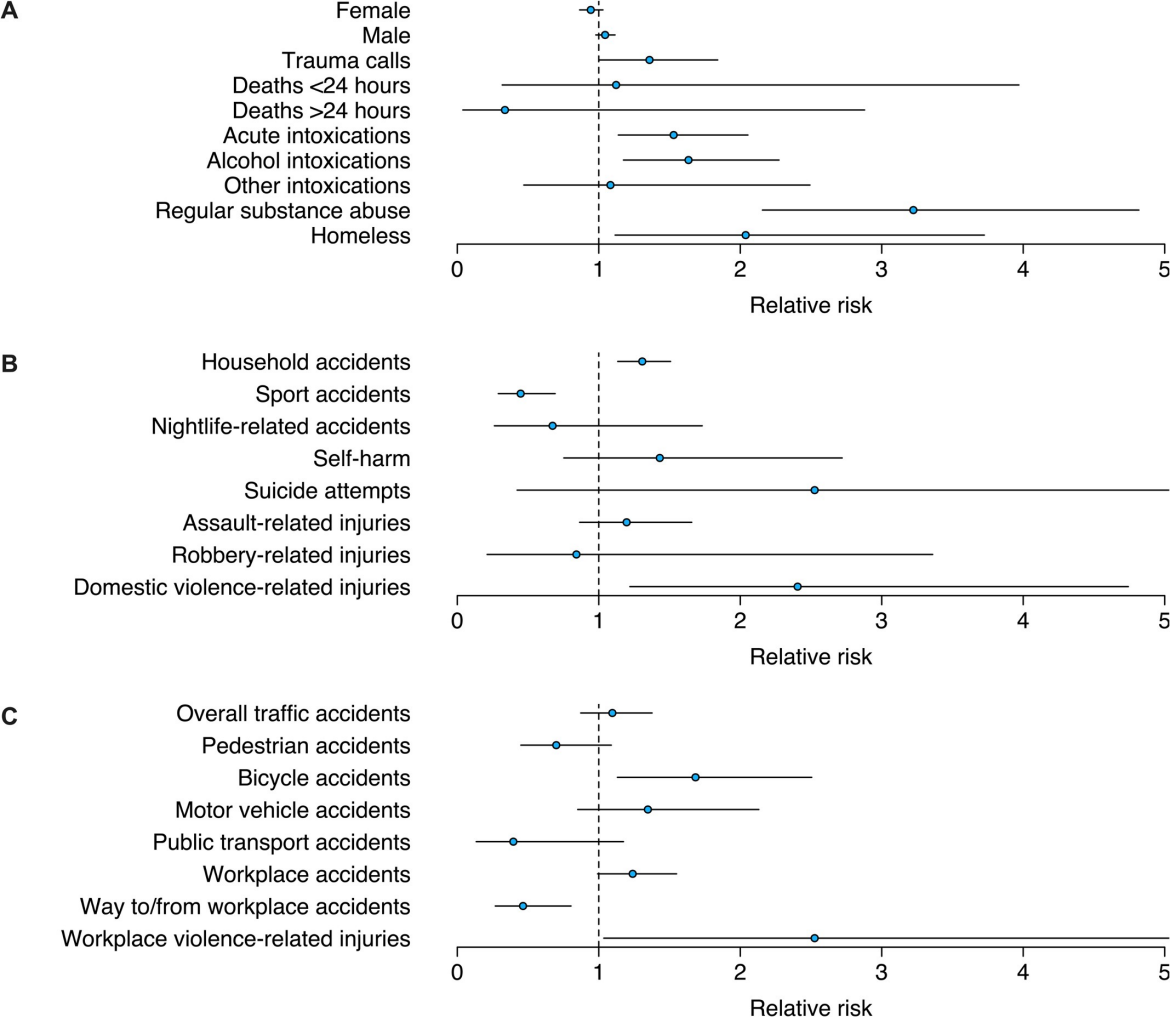
Plots showing RRs during the SHUTDOWN compared to the CTRL period for selected outcomes. (A) Demographic specifics, trauma calls, deaths and substance abuse, (B) trauma causes within private environments and (C) within traffic and workplace environments. Circles indicate RRs (SHUTDOWN vs. CTRL) and whiskers the 95% CIs.

Incidence proportions increased for acute intoxications with an RR of 1.53 (SHUTDOWN vs. CTRL, 95% CI: [1.14; 2.05]). Most intoxications resulted from alcohol intake, where incidence proportions also increased during the SHUTDOWN period with an RR of 1.63 (SHUTDOWN vs. CTRL, 95% CI: [1.17; 2.27]), while no relative difference was seen for other intoxications. Regular substance abuse increased in absolute and relative numbers during the SHUTDOWN period with an RR of 3.22 (SHUTDOWN vs. CTRL, 95% CI: [2.16; 4.82]). Incidence proportions of patients that reported to be homeless increased during the SHUTDOWN compared to the CTRL period with an RR of 2.04 (SHUTDOWN vs. CTRL, 95% CI: [1.12; 3.73]) (Fig 3A).

### Accidents/injuries in private environments

Incidence proportions of household accidents increased with an RR of 1.31 (SHUTDOWN vs. CTRL, 95% CI: [1.13; 1.51]) while sport accident incidence proportions decreased with an RR of 0.45 (SHUTDOWN vs. CTRL, 95% CI: [0.29; 0.69]).

No relevant changes were observed for incidence proportions of nightlife-related accidents, injuries resulting from self-harm, suicide attempts, assault-related injuries and robbery-related injuries comparing both periods. Increased incidence proportions were, however, observed for domestic violence-related injuries during the SHUTDOWN compared to the CTRL period with an RR of 2.41 (SHUTDOWN vs. CTRL, 95% CI: [1.22; 4.74]) (Fig 3B).

### Traffic and workplace accidents/injuries

Incidence proportions of overall traffic-related accidents remained almost constant in the two periods assessed. Similarly, incidence proportions of accidents involving motor vehicles, pedestrians and public transport did not differ between study periods. In contrast, incidence proportions of injured bicycle passengers increased during the SHUTDOWN compared to the CTRL period with an RR of 1.68 (SHUTDOWN vs. CTRL, 95% CI: [1.13; 2.51]).

While the relative number of injuries sustained on the way to or from work decreased during the SHUTDOWN period with an RR of 0.46 (SHUTDOWN vs. CTRL; 95% CI: [0.27; 0.80]), there were no differences in incidence proportions of injuries sustained at work. Injuries originating from violence at work increased on a relative scale during the SHUTDOWN compared to the CTRL period with an RR of 2.53 [1.04; 6.16] (Fig 3C).

### Non-traumatic orthopedic presentations

**Fig 4:**
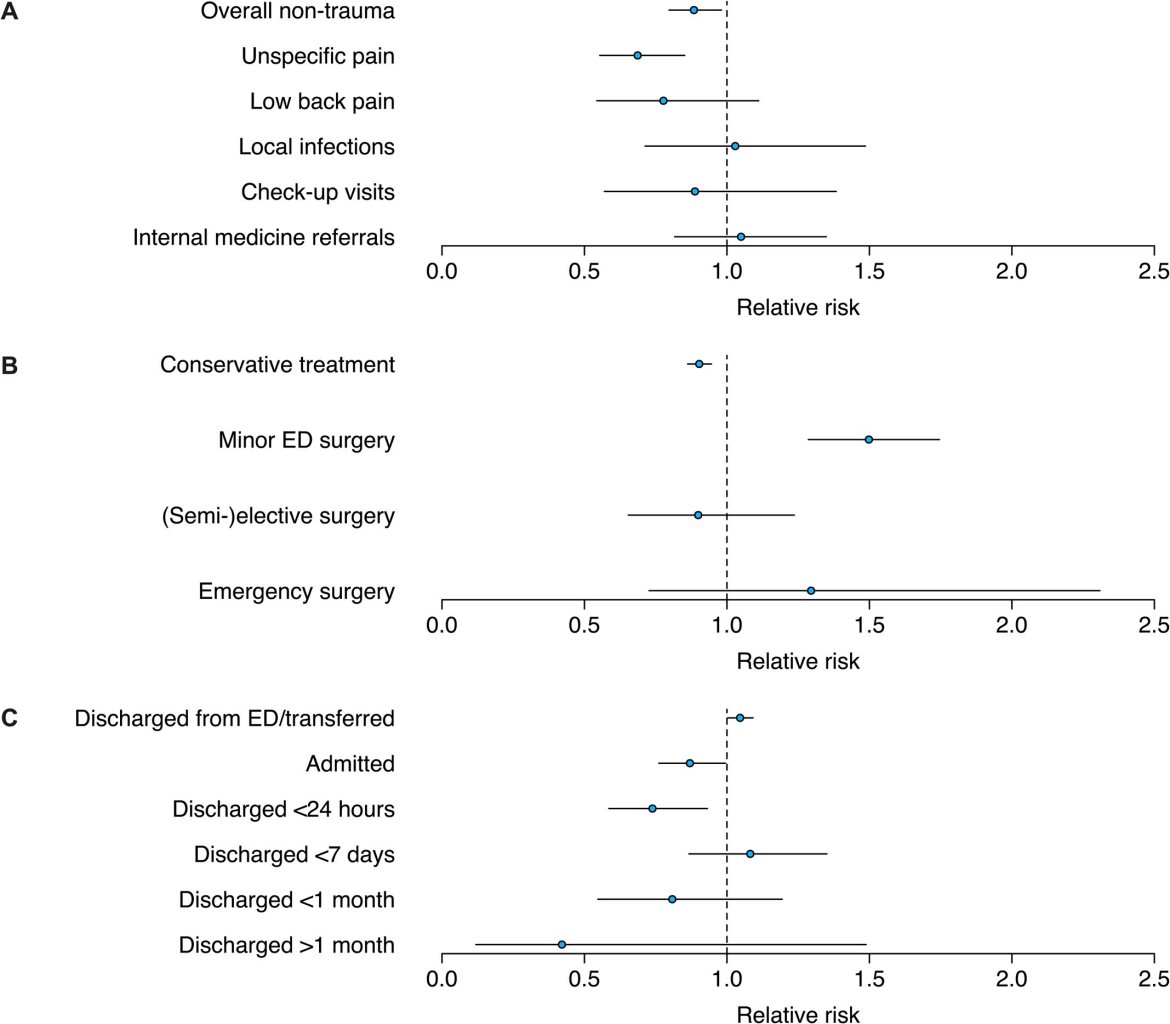
Plots showing RRs during the SHUTDOWN compared to the CTRL period for selected outcomes. (A) Non-traumatic orthopedic visits, (B) treatment and (C) discharge metrics. Circles indicate RRs (SHUTDOWN vs. CTRL) and whiskers the 95% CIs. ED = emergency department.

Incidence proportions of patients presenting to the ED with non-traumatic orthopedic symptoms decreased during the SHUTDOWN period with an RR of 0.88 (SHUTDOWN vs. CTRL; 95% CI: [0.80; 0.98]). More specifically, incidence proportions of patients presenting with unspecific pain decreased with an RR of 0.69 (SHUTDOWN vs. CTRL; 95% CI: [0.55; 0.85]) during the SHUTDOWN compared to the CTRL period. Numbers of other non-traumatic orthopedic presentations, including low back pain, local infections, check-up visits and cases with an internal medicine focus and a subsequent referral to internal medicine, remained without relative differences during respective periods (Fig 4A).

### Treatment, admissions and discharges

Incidence proportions of conservatively treated patients decreased during the SHUTDOWN compared to the CTRL period with an RR of 0.90 (SHUTDOWN vs. CTRL, 95%CI: [0.86; 0.95]), while minor ED surgery incidence proportions increased with an RR of 1.50 (SHUTDOWN vs. CTRL, 95% CI [1.29; 1.75]). Incidence proportions of (semi)-elective surgeries and emergency surgeries remained relatively unchanged during respective periods. Finally, incidence proportions of admitted patients and discharged patients <24 hours decreased with an RR of 0.87 (SHUTDOWN vs. CTRL, 95% CI: [0.76; 0.99]) and 0.74 (SHUTDOWN vs. CTRL, 95% CI: [0.59; 0.93]), respectively, during the SHUTDOWN compared to the CTRL period (Fig 4B and 4C). Numbers are summarized in Table 2.

### Fractures and intracranial hemorrhages

**Fig 5:**
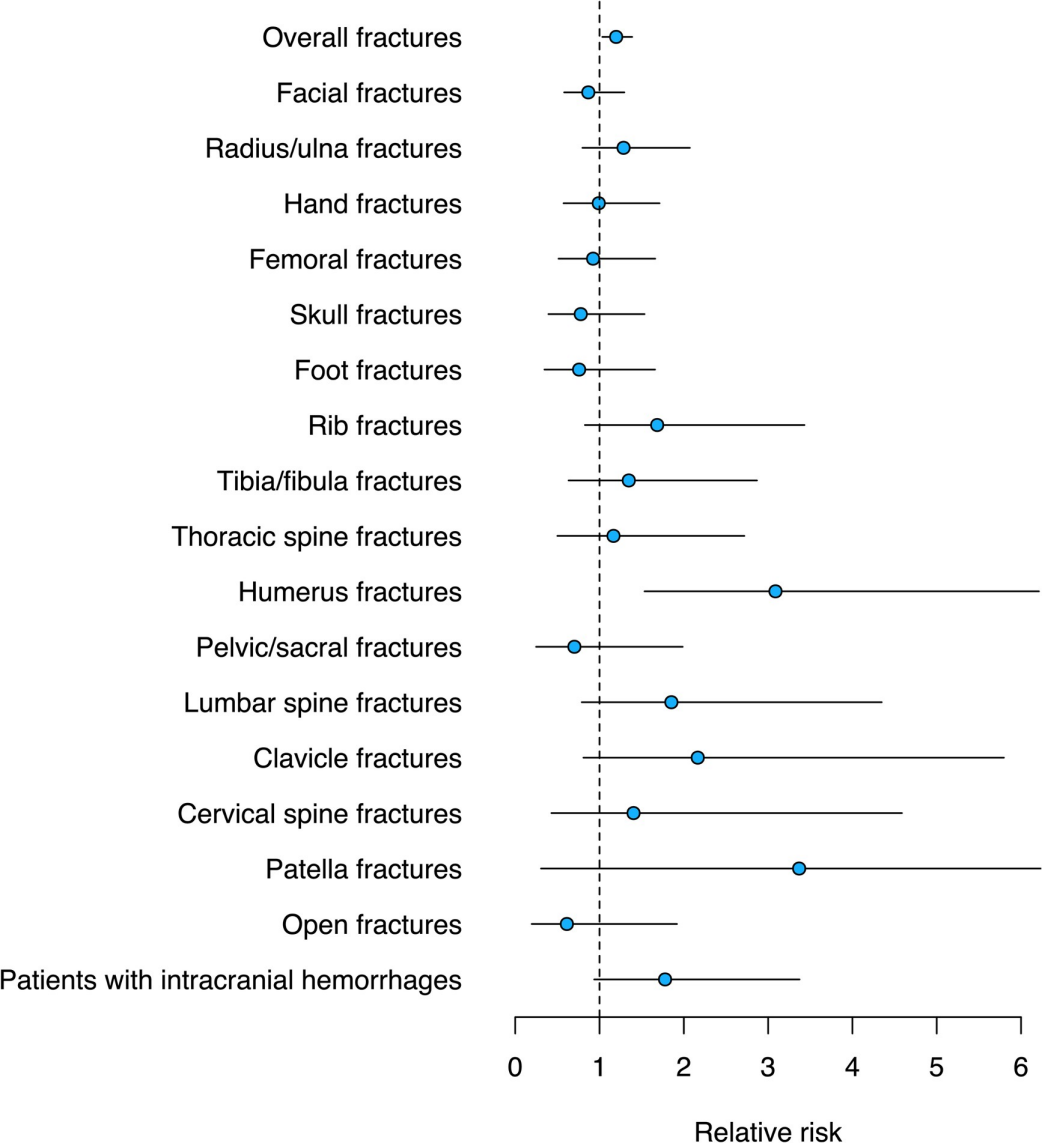
Plot showing RRs during the SHUTDOWN compared to the CTRL period for fractures and patients with intracranial hemorrhages. Circles indicate RRs (SHUTDOWN vs. CTRL) and whiskers the 95% CIs. Numbers are presented in Table 3.

Overall fracture incidence proportions increased during the SHUTDOWN period, most pronouncedly for humerus fractures where the RR was 3.09 (SHUTDOWN vs. CTRL, 95% CI: [1.53; 6.21]) (Fig 5). Incidence proportions and RRs of skull, facial, cervical spine, radius/ulna, hand, clavicle, thoracic spine, rib, pelvic/sacral, lumbar, femoral, tibia/fibula, patella, foot and open fractures remained similar during the SHUTDOWN and CTRL period. Absolute numbers, incidence proportions and RRs for fractures and patients with intracranial hemorrhages are shown in Fig 5 and Table 3.

### COVID-19 testing

During the PRE period 402 patients were tested for COVID-19 in our ED. Of these, five test results were positive (1.2%). During the SHUTDOWN period 905 patients were tested for COVID-19 in the ED. Of these, 83 test results were positive (9.2%). During the POST period our ED tested 1042 patients for COVID-19. Of these, 14 test results were positive (1.3%). Of note, testing criteria changed between February and May 2020 and especially in the POST period also asymptomatic patients were tested before ward admission.

## Discussion

In the current study we describe the impact of the COVID-19 shutdown on patient numbers in an academic Level I Trauma Center ED in Berlin, Germany, by comparison to three other periods before (CTRL, PRE) and after (POST) the shutdown (SHUTDOWN). For the CTRL and SHUTDOWN periods, we analyzed trauma causes and injury patterns in detail.

Compared to the CTRL period in 2019, daily numbers of orthopedic trauma cases declined by nearly 15% during the PRE and by over 40% during the SHUTDOWN period. Correspondingly, an increase of 17% was observed during the POST compared to the SHUTDOWN period. The u-shaped development of ED and orthopedic trauma cases over time displays a gradual decrease and slow increase in patient numbers during the COVID-19 shutdown period. U-shaped recoveries were also described for other sectors, like aviation and trade during the COVID-19 pandemic [20, 21]. In case of further COVID-19 related shutdowns in the future, the longitudinal development of ED and orthopedic trauma cases could turn into a w-shape [21].

As previously reported, trauma admissions show seasonal differences with a mid-year peak and the “trauma season” spanning from April to November [22]. Although falls and motor vehicle accidents were reported to be more frequent in the winter season [23], trauma numbers in March/April were comparable to those in February/March in ordinary years without a pandemic [24]. This allows the assumption that lower orthopedic trauma cases during the PRE compared to the CTRL period, may have been caused by people already being more cautious during the early months of the COVID-19 pandemic, although a shutdown was not yet in place.

Our findings go in accordance with a previous report from the Midland trauma registry in New Zealand [10]. The group found a 43% decrease of injury-related admission numbers during the national COVID-19 lockdown compared to a control period in 2019 in a Level I Trauma Center. In their study, no statistical analyses could be performed on injury patterns due to a low number of included patients (n = 195) [10]. In contrast, we compared 1082 orthopedic trauma cases during the SHUTDOWN in 2020 with 1822 cases during the CTRL period in 2019 regarding trauma causes and injury patterns. The observation of a relevant increase in domestic violence confirms concerns that were previously raised by other authors regarding intimate partner violence during the COVID-19 pandemic and concomitant shutdown environments [25–27]. A suspected high number of unreported cases of intimate partner violence further underlines the relevance of this finding [28]. Concrete social support plans are warranted during quarantine and shutdown scenarios to protect vulnerable individuals and families in potential future pandemics and related shutdowns [29]. Recent data from the United Kingdom indicate that inaccessibility to social support during the COVID-19 pandemic led to a reduction in well-being and increased anxiety in the elderly [30]. The importance of a timely response to mental health needs has been described for previous pandemics [31] and can also be seen in the increased workplace violence-related injuries observed during the SHUTDOWN period in this study, which might have been caused by psychological stress due to fear of going into work as essential workers despite a global pandemic and a concomitant risk of infection. Effective psychological support can also be provided by non-psychiatric support groups, if mental health professionals are scarce and training is available [31].

Our data disclose higher rates of acute and regular substance abuse in orthopedic trauma patients during the SHUTDOWN compared to the CTRL period. This is in accordance with observations from the Hubei province in China during the COVID-19 lockdown, where an increase in hazardous and harmful alcohol use during the COVID-19 lockdown was observed [32]. Similarly, data from the 2003 SARS epidemic in China pointed toward a risk of alcohol dependence and abuse during infectious disease outbreaks [33].

We demonstrated that injuries resulting from self-harm and suicide attempts did not differ between the two periods assessed. This was expected as suicide numbers seem to rise only after a certain delay following catastrophic events [34]. Increased suicide rates after economic crises [35, 36] and natural disasters [34] have been well-documented, and a rise in suicide numbers in the months and years following the COVID-19 pandemic is anticipated [37]. Tele-counselling alongside 24/7 crisis response services for emotional, mental and behavioral support have been suggested and implemented as tools to tackle COVID-19-related suicide intentions [38, 39]. Further, continuous and transparent communication between healthcare officials, governments and society may decrease anxiety and create sustainable information structures in times shaped by uncertainty and fear [40, 41].

We observed that not only the total number of ED cases decreased, but also the number of ED visits, that were likely unrelated to the COVID-19 shutdown itself, including low back pain and unspecific pain. This finding may be explained by a tendency to seek less medical aid during pandemic-related shutdowns as recently demonstrated in a retrospective analysis from 15 Italian cardiovascular centers [42]. In their study, the authors described a reduction in acute coronary syndrome-related hospital admissions during the COVID-19 lockdown in Italy. They observed markedly elevated mortality rates not fully explained by SARS-CoV-2 infections during the same period. The group hypothesized that a relevant number of deaths from acute coronary syndrome may have occurred unnoticed during the COVID-19 lockdown in Italy, as patients did not seek medical assistance [42]. In this study we found an absolute and relative decrease in the number of patients presenting with household-related injuries during the SHUTDOWN period. As people spent more time at home during shutdowns, it is unlikely that the number of household-related injuries decreased. Yet, if people with life-threatening conditions like acute coronary syndrome were less likely to seek medical care when experiencing symptoms during the COVID-19 pandemic, we speculate that this could also be the case for patients with less severe conditions. Whether these patients were in fact not in need of medical help provided by ED services or will require medical support at a later stage (e.g. for infections, missed fractures) remains speculative. This finding however raises questions about the potential prevention of ED visits due to non-urgent and chronic conditions and could help to educate people on alternatives to ED care in those cases [43]. Data from a tertiary trauma center in Spain reported no differences in the number of osteoporotic hip fractures between the period of March 14^th^ to April 2^nd^ 2020 (n = 36) and a control period in 2019 (n = 43) [11]. In opposition to this, two trauma institutions in Italy observed a relevant decrease in femoral fracture numbers during a two-month period from February 22^nd^ to April 18^th^ 2020 (n = 121) compared with the previous year (n = 169) [44]. Our data showed an absolute but not a relative decrease in femoral fractures during the SHUTDOWN (n = 17) compared to the CTRL (n = 31) period. Reasons for such a decline in fractures, which are mainly sustained at home or in nursing homes, remain hypothetical and although our hospital is considered a central treatment facility for COVID-19 patients, a shift in patient distribution with femoral fractures to other hospitals by emergency and first responder services was not confirmed by the services. Social distancing and contact restrictions were also implemented in nursing homes, which led to reduced community activities and by that a potentially reduced risk of falling.

Although the number of overall fractures decreased, we observed an absolute and relative increase of humerus fractures. Data from Italy showed an overall decrease of 65% for shoulder and elbow trauma during March and April 2020 compared to the same period in 2019, yet the prevalence of proximal humerus fractures did not differ between the two periods. The vast majority of cases was caused by an accidental fall at home [45]. As people spent more time at home during the COVID-19 shutdown, this may explain why the number of this specific injury was found to be increased in our study.

Our data show that with an overall decline in orthopedic trauma patients, the absolute number of patients requiring surgical care decreased as well. These findings are particularly relevant for resource planning and logistics during potential future pandemics and shutdowns. In our academic Level I Trauma Center ED in Berlin, Germany, the reallocation of orthopedic trauma healthcare providers and operating theatre staff to high maintenance COVID-19 wards was largely discussed and well-considered prior to the start of the pandemic in Berlin, Germany. According to our data, these strategies proved useful and justifiable with regards to the numbers of orthopedic trauma cases.

Limitations of this study are its retrospective and monocentric character as well as the lack of adjustments for potential confounding factors. While we were able to analyze a great number of orthopedic trauma cases in one of Europe’s largest university hospitals, differences in absolute numbers and incidence proportions should always be compared to results from other large trauma institutions to validate the findings. We hope these data will aid future national and international pandemic plans regarding ED service assessments and the distribution of supporting social services.

## Supporting information

S1 File List of variables for orthopedic trauma patients.
(DOCX)

S1 Data (XLSX)
