# Supplementary material for: Impact of the COVID-19 shutdown on orthopedic trauma numbers and patterns in an academic Level I Trauma Center in Berlin, Germany
Source: PLoS One. 2021 Feb 16;16(2):e0246956. doi: 10.1371/journal.pone.0246956 (PMC7886210; doi:10.1371/journal.pone.0246956)
Supplement: S1 File — List of variables for orthopedic trauma patients. (DOCX) [file pone.0246956.s001.docx]

**S1 File. List of variables for orthopedic trauma patients:** Sex, trauma calls, deaths <24 hours, deaths >24 hours, acute intoxications, alcohol intoxications, other intoxications, regular substance abuse, homelessness, household accidents, sport accidents, nightlife-related accidents, self-harm, suicide attempts, assault-related injuries, robbery-related injuries, domestic violence-related injuries, overall traffic accidents, pedestrian accidents, bicycle accidents, motor vehicle accidents, public transport accidents, workplace accidents, way to/from workplace accidents, workplace violence-related injuries, overall non-trauma, unspecific pain, low back pain, local infections, check-up visits, internal medicine referrals, conservative treatment, minor ED surgery, (semi-)elective surgery, emergency surgery, discharged from ED/transferred, admitted, discharged <24 hours, discharged <7 days, discharged <1 month, discharged >1 month, overall fractures, facial fractures, radius/ulna fractures, hand fractures, femoral fractures, skull fractures, foot fractures, rib fractures, tibia/fibula fractures, thoracic spine fractures, humerus fractures, pelvic/sacral fractures, lumbar spine fractures, clavicle fractures, cervical spine fractures, patella fractures, open fractures, patients with intracranial hemorrhages.
